# Single-cell RNA sequencing reveals the dysfunctional characteristics of PBMCs in patients with type 2 diabetes mellitus

**DOI:** 10.3389/fimmu.2024.1501660

**Published:** 2025-01-23

**Authors:** Jindong Zhao, Zhaohui Fang

**Affiliations:** ^1^ Department of Endocrinology Two, The First Affiliated Hospital of Anhui University of Chinese Medicine, Hefei, Anhui, China; ^2^ Center for Xin’an Medicine and Modernization of Traditional Chinese Medicine of IHM, The First Affiliated Hospital of Anhui University of Chinese Medicine, Hefei, Anhui, China; ^3^ Diabetes Institute, Anhui Academy Chinese Medicine, Hefei, China

**Keywords:** type 2 diabetes mellitus, peripheral blood mononuclear cells, single-cell RNA sequencing, T cells, monocytes

## Abstract

**Introduction:**

Type 2 diabetes mellitus (T2DM) is a disease that involves autoimmunity. However, how immune cells function in the peripheral blood remains unclear. Exploring T2DM biomarkers via single-cell RNA sequencing (scRNA-seq) could provide new insights into the underlying molecular mechanisms.

**Methods:**

The clinical trial registration number is ChiCTR2100049613. In this study, we included three healthy participants and three T2DM patients. The observed clinical indicators included weight and fasting blood glucose (FBG), glycosylated haemoglobin A1c (HbA1c) and fasting insulin levels. Direct separation and purification of peripheral blood mononuclear cells (PBMCs) were performed via the Ficoll density gradient centrifugation method. Immune cell types were identified via scRNA-seq. The differentially expressed genes, biological functions, cell cycle dynamics, and correlations between blood glucose indicators and genes in different cell types were analysed.

**Results:**

There were differences between the healthy and T2DM groups in terms of FBG and HbA1c (p<0.05 or p<0.01). We profiled 13,591 cells and 3188 marker genes from PBMCs. B cells, T cells, monocytes, and NK cells were grouped into 4 subclusters from PBMCs. CD4+ T cells are mainly in the memory activation stage, and CD8+ T cells are effectors. Monocytes include mainly CD14+ monocytes and FCGR3A+ monocytes. There were 119 differentially expressed genes in T cells and 175 differentially expressed genes in monocytes. Gene set enrichment analysis revealed that the marker genes were enriched in HALLMARK_ INTERFERON_GAMMA_RESPONSE and HALLMARK_TNFA_SIGNALING_VIA_ NFKB. Moreover, TNFRSF1A was identified as the core gene involved in network interactions in T cells.

**Discussion:**

Our study provides a transcriptional map of immune cells from PBMCs and provides a framework for understanding the immune status and potential immune mechanisms of T2DM patients via scRNA-seq.

**Clinical trial registration:**

http://www.chictr.org.cn, identifier ChiCTR2100049613.

## 1 Introduction

Diabetes mellitus (DM) is a metabolic disease characterized by hyperglycaemia, and it can be caused by genetic factors, environmental factors, and autoimmune factors, among others. To date, four distinct types of DM have been defined, of which type 1 DM (T1DM) is mostly an autoimmune disease. T lymphocytes are activated *in vivo* and cause rapid destruction and functional failure of islet beta cells, leading to the development of T1DM (1, 2). However, type 2 DM (T2DM) accounts for approximately 90–95% of DM cases, with an incidence of 11.2% in China (3). T2DM comprises a group of heterogeneous diseases whose complex pathogenesis has not been fully elucidated (4). The pathogenesis of T2DM is related mainly to insulin resistance (IR), which leads to prediabetes and ultimately DM.

Blood glucose is the main biomarker used for the diagnosis of T2DM. The discovery of other early biomarkers or molecular, pathological, and immunological changes is important for improving the diagnosis and evaluation of T2DM (5). To date, the phenotypes and roles of T cells, NK cells, monocytes and other immune cells have received less attention than those of other systems involved in DM (6, 7). In recent years, increasing evidence has shown that immune disorders are the main factors involved in the occurrence of T2DM (8–10). Single-cell RNA sequencing (scRNA-seq) is a new technique that can be used to elucidate cell heterogeneity and quantify the expression profiles of individual genes in individual cells, making it easier to study the roles of specific genes. scRNA-seq can be used to elucidate specific functional alterations in cells that may reveal cellular phenotypes and heterogeneity and to identify biomarkers for the diagnosis and treatment of T2DM; these biomarkers may also help predict outcomes and complications in individual cases (11). The role of islet cell types in the genetic signalling pathways associated with T2DM susceptibility, particularly the role of islet beta cell specificity, have been investigated (12, 13). Huang Y et al. detected 6 islet cell types and reported that SLC2A2, SERPINF1, RASGRP1 and CHL1 are biomarkers of T2DM that can be used for clinical diagnosis (14). Lee H et al. reported that CD8+ effector T cells in the peripheral blood mononuclear cells (PBMCs) of patients with T2DM had a reduced cytotoxicity score and a heightened level of exhaustion (15).

**Figure 1:**
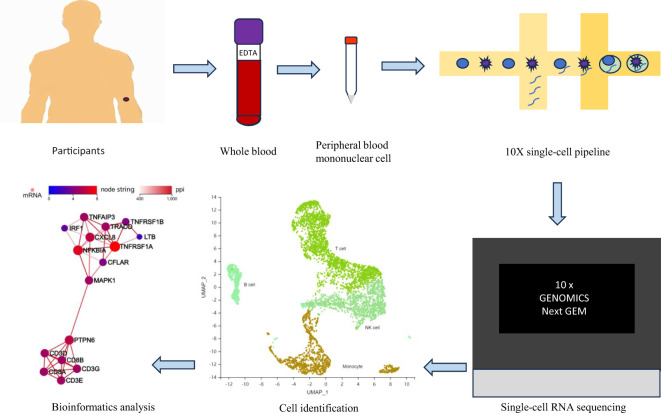
Research flow chart. EDTA, ethylene diamine tetraacetic acid.

In this study, we obtained scRNA-seq data from the whole blood of healthy participants and T2DM patients by labelling single-cell clusters and identifying key cell clusters via typical gene expression levels to understand the expression of genes in every cell and the communication between cells. This analysis may provide new insights into a framework for understanding the immune status of T2DM patients (**Figure 1**).

## 2 Materials and methods

### 2.1 Experimental samples

This study was a prospective, controlled trial aimed at analysing the possible immune mechanisms in T2DM patients. The study was registered as a Chinese clinical trial on the WHO international clinical trial registry platform (ChiCTR2100049613). All methods were carried out in accordance with the CONSORT statement. All experimental protocols were approved by the Ethics Committee of the First Affiliated Hospital of Anhui University of Traditional Chinese Medicine. The ethics approval number is 2021AH-39. All participants provided written informed consent before participating. Three healthy participants and three patients with T2DM were included.

### 2.2 Participant

Patients were diagnosed with T2DM according to the guidelines for the prevention and treatment of T2DM in China (2020 edition). The diagnostic criteria for healthy participants were the absence of a history of systemic disease, such as hypertension, T2DM, or cardiopulmonary insufficiency, and no use of systemic or topical medications. The inclusion criteria for T2DM patients were a glycosylated haemoglobin A1c (HbA1c) level ≤ 7.5%, course of T2DM disease ≤ 3 years, and aged 18–70 years; both sexes were included. The participants were informed of the study procedures and voluntarily signed an informed consent form.

The exclusion criteria for T2DM patients were the presence of T1DM, gestational DM, T2DM requiring insulin therapy, or other special types of DM; acute complications of T2DM; severe cardiovascular and cerebrovascular diseases; severe primary diseases, such as liver, kidney and haematopoietic system diseases; allergy to the known ingredients of the study drug or chronic allergies; pregnancy, lactation, having recently given birth, or planning to become pregnant; long-term alcoholism, drug dependence or mental illness; and participation in another drug clinical trial within one month before the screening period for this study. Finally, all participants were deemed suitable for participation in this clinical study according to the opinion of the investigator.

### 2.3 Observation indices

**Figure 2:**
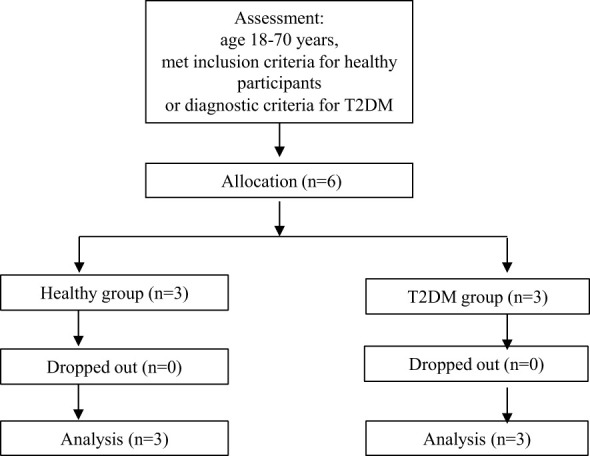
CONSORT participant flow chart. T2DM, type 2 diabetes mellitus.

The collected background information included age, sex, height, weight, etc. Fasting blood glucose (FBG), alanine aminotransferase, aspartate transaminase, blood urea nitrogen, and creatinine levels were determined with a Beckman AU5800 fully automatic biochemical analyser (Beckman, US). The level of HbA1c was determined with a Bio-Rad D-100 HbA1c analyser (Bio-Rad, US). The level of fasting insulin (FINS) was determined with an AutoLumo A2000 Plus fully automatic chemiluminescence analyser (Autobio, Zhengzhou, China). The homeostasis model assessment–IR (HOMA–IR) score was calculated as FINS (μIU/ml) × FBG (mmol/L) ÷ 22.5 (16). Safety indicators included blood pressure, heart rate, routine blood tests, and routine urine tests (**Figure 2**).

### 2.4 scRNA-seq

ScRNA-seq was performed by BGI Shenzhen. The 10x Genomics Chromium allows high-throughput single-cell 3’ mRNA quantitative analysis. Afterwards, 5 mL of whole blood containing ethylene diamine tetraacetic acid was added to a 15 mL centrifuge tube with 3 mL of Ficoll lymphocyte separation media. An equal volume of 1X PBS was added to each blood sample. The diluted blood samples were layered carefully in Ficoll lymphocyte separation liquid and then centrifuged at 400 × g at 18–20°C for 30 min continuously. The mononuclear cell layer was transferred to a 15 mL sterile centrifuge tube with a sterile pipette. Three volumes of 1× PBS were added to the lymphocyte layer, which was carefully mixed via pipetting. The samples were subsequently centrifuged at 400 × g, after which the supernatant was discarded. Then, 6 mL of 1× PBS was added to the lymphocyte layer, which was again carefully mixed via pipetting. The mixture was subsequently centrifuged at 400 × g for 10 min, and the supernatant was discarded. The cells were resuspended in the desired volume of 1× PBS and stained with 0.4% trypan blue. Samples with greater than 80% cell viability were used for library construction. The prepared single-cell suspensions were subsequently partitioned into gel beads in emulsion in an automated Chromium Controller, after which the mRNAs were reverse transcribed into cDNAs. The reaction system was configured in sequence for breaking gel beads in emulsion, cDNA amplification, fragmentation, end repair, A-tailing, and adaptor ligation polymerase chain reaction. After reacting at a suitable temperature for a fixed period, the products were separately purified in an appropriately configured reaction system. After library quality control, single-stranded polymerase chain reaction products were produced via denaturation. Single-stranded cyclized products were produced with a circularization reaction system. Single-stranded circular DNA molecules were replicated, and a DNA nanoball that contained multiple copies of DNA was generated. DNA nanoballs of sufficient quality were loaded into patterned nanoarrays and sequenced through combinatorial probe–anchor synthesis.

The raw gene expression matrix generated from each sample was aggregated via Cell Ranger (v5.0.1) (17), which is provided on the 10x Genomics website. Downstream analysis was performed with the R package Seurat (v 3.2.0) (18). Specifically, cells with fewer than 200 genes or with > 90% of the proportion of the maximum genes were filtered. For the mitochondrial metric, the cells were sorted in descending order of the mitochondrial read ratio, and the top 15% of the cells were filtered. Potential doublets were identified and removed via doublet detection (19). Cell cycle analysis was performed with the cell cycle scoring function of the Seurat program. The gene expression dataset was normalized. Uniform manifold approximation and projection (UMAP) was subsequently used for two-dimensional visualization of the resulting clusters. For each cluster, marker genes were identified with the FindAllMarkers function as implemented in the Seurat package (V3.2.0, logFC > 0.25, minPct > 0.1 and Padj ≤ 0.05). The clusters were then marked as known cell types via the scRNA-seq atlas method (20). Differentially expressed genes (DEGs) across different samples were identified with the FindMarkers function (logFC > 0.25, minPct > 0.1 and Padj ≤ 0.05). Volcano plots were created with the ggplot2 package. The threshold for the log fold change was set at 0.2, and that for p values was set at 0.05. Gene Ontology (GO) analysis was performed via the phyper function of the R package (R-3.1.1). Kyoto Encyclopedia of Genes and Genomes (KEGG, V93.0) enrichment analysis results. GO and KEGG pathways with p or Q values ≤0.05 were considered significantly enriched (21).

### 2.5 Genetic evidence calculator

The Type 2 Diabetes Knowledge Portal (https://t2d.hugeamp.org/) contains summary data on genetic correlations, genome annotations, bioinformatics results, expertise in T2DM and related traits, blood glucose, etc. The human genetic evidence calculator integrates several kinds of human genetic results to quantify genetic support for the involvement of a gene in a disease or phenotype of interest (22).

### 2.6 Statistical analysis

The statistical analyses were conducted in SPSS 23.0. Continuous variables are expressed as the means ± standard deviations. Categorical variables are presented as numbers or percentages. Two-group comparisons were conducted via independent-samples t tests or chi-square tests. A p value less than 0.05 was considered to indicate statistical significance.

## 3 Results

### 3.1 Comparison of clinical baseline information

There were no significant differences in age, sex, or disease course between the two groups, but there were significant differences in body mass index (BMI) or FBG and HbA1c levels between the two groups, as shown in **Table 1**.

**Table 1:** Baseline clinical information.

Characteristic	Healthy group (n = 3)	T2DM group (n = 3)
Age (years)	56.33 ± 16.74	48.33 ± 7.02
Sex (male/female)	2/1	2/1
Course of disease (years)	0.00 ± 0.00	0.56± 0.42
BMI (kg/m^2^)	22.74 ± 2.56	27.97 ± 0.76^*^
FBG (mmol/L)	5.36 ± 0.49	7.21 ± 0.15^**^
HbA1c (%)	5.47 ± 0.25	6.15 ± 0.29^*^
FINS (μIU/ml)	7.62±2.93	14.85±9.31
HOMA–IR	1.78±0.54	4.72±2.90

^*^ P < 0.05, ^**^ P < 0.01 compared with the healthy group. FBG, fasting blood glucose; BMI, body mass index; HbA1c, glycosylated haemoglobin A1c; FINS, fasting insulin; HOMA–IR, homeostatic model assessment of insulin resistance.

### 3.2 Single-cell clustering and cell type identification

**Figure 3:**
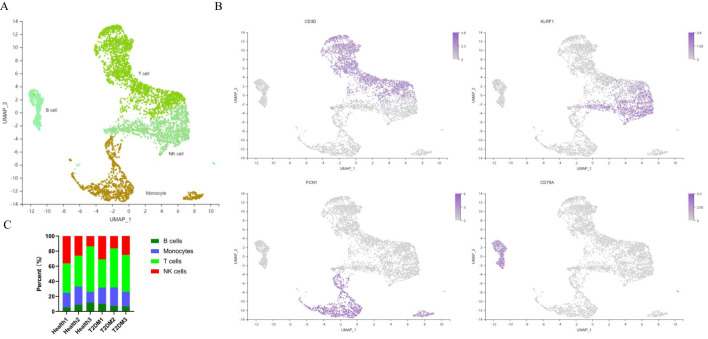
Cell atlas of immune infiltrates in PBMCs. **(A)** UMAP plot of immune cell clusters. **(B)** Classic marker genes for each cell type. **(C)** Each sample corresponds to the cell type in each cluster. PBMCs, peripheral blood mononuclear cells; UMAP, uniform manifold approximation and projection. n=3 in each group.

After quality control and filtering, 13,591 cells remained for analysis. Unsupervised clustering of single-cell data after normalization and aggregation was performed via Seurat 3.2.0. Four cell types were identified (**Figure 3A**). We annotated the cell types according to the expression of classic marker genes, and the classic genes that were differentially expressed in those cells were consistent with the annotations (**Figure 3B**). The percentages of the 4 cell types in patients with T2DM and healthy participants are shown in **Figure 3C**.

**Figure 4:**
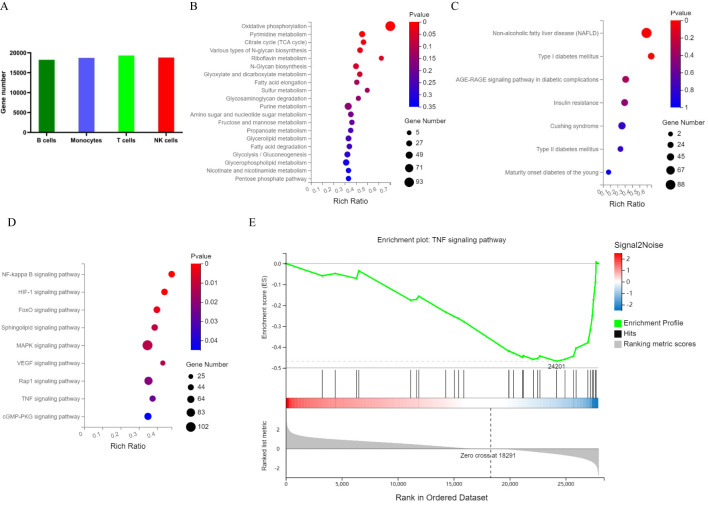
Map of PBMC genes and marker genes in T2DM patients. **(A)** The number of genes in each cluster. **(B)** KEGG metabolic pathways enriched with marker genes. **(C)** KEGG pathways enriched in marker genes of endocrine and metabolic diseases. **(D)** KEGG pathways enriched in marker genes of signal transduction pathways. **(E)** GSEA of PBMCs by marker genes. PBMCs, peripheral blood mononuclear cells; KEGG, Kyoto Encyclopedia of Genes and Genomes; GSEA, gene set enrichment analysis. n=3 in each group.

A total of 20,092 genes were identified in the 6 samples. Among the 4 cell types, T cells expressed the most genes (**Figure 4A**). A total of 3188 marker genes in the two groups were annotated to the KEGG metabolic pathway (**Figure 4B**). According to the KEGG pathway term level 2, 7 endocrine and metabolic diseases and 9 signal transduction pathways were screened (**Figures 4C, D**). In contrast, the presence of 11 marker genes, including PKM, MAPK1, MAPK3, PIK3R1, HK1, HK3, INSR, PIK3CD, SOCS1, IRS2 and TNF, was associated with T2DM status. Pathways with significant differences included the NF-κB, HIF-1 and TNF signalling pathways. Notably, the gene set enrichment analysis (GSEA) results also revealed the pathways with the greatest differences in the TNF signalling pathway (**Figure 4E**).

### 3.3 Clustering and subtype analysis of T cells

**Figure 5:**
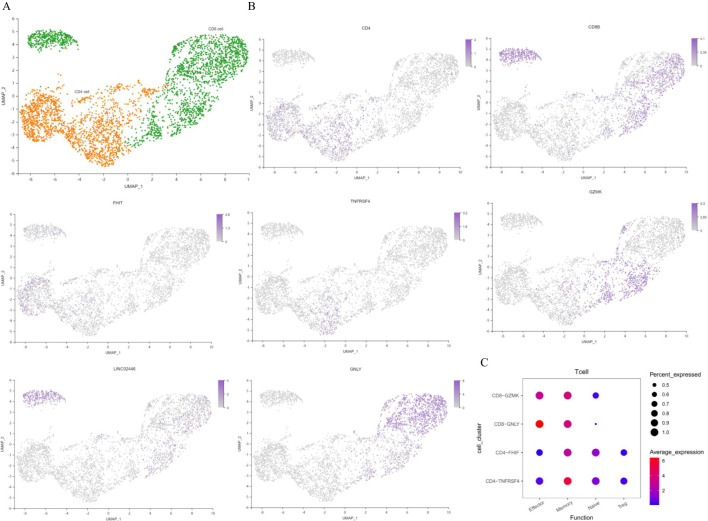
Characterization of T cells. **(A)** UMAP plot of T cells. **(B)** Feature map showing the marker genes for various cell types. **(C)** Dot plot of representative activation stage signatures in T-cell clusters. UMAP, uniform manifold approximation and projection. n=3 in each group.

T cells are the main specific immune cells found in patients with T2DM. Unsupervised clustering of T cells revealed two CD4+ T-cell clusters (including 3127 cells) and three CD8+ T-cell clusters (including 3678 cells) (**Figure 5A**). T cells were annotated separately by canonical genes, and the expression of the canonical genes of these cell types was consistent with the annotation (**Figure 5B**). There were 387 marker genes found in CD4+ T cells and 684 marker genes in CD8+ T cells. The genes in CD4+ and CD8+ T cells were analysed, and the transcription signal score was calculated. The results suggested that CD4+ T cells in T2DM patients tended to be in memory and naïve states, whereas CD8+ T cells tended to be in effector and memory states (**Figure 5C**).

**Figure 6:**
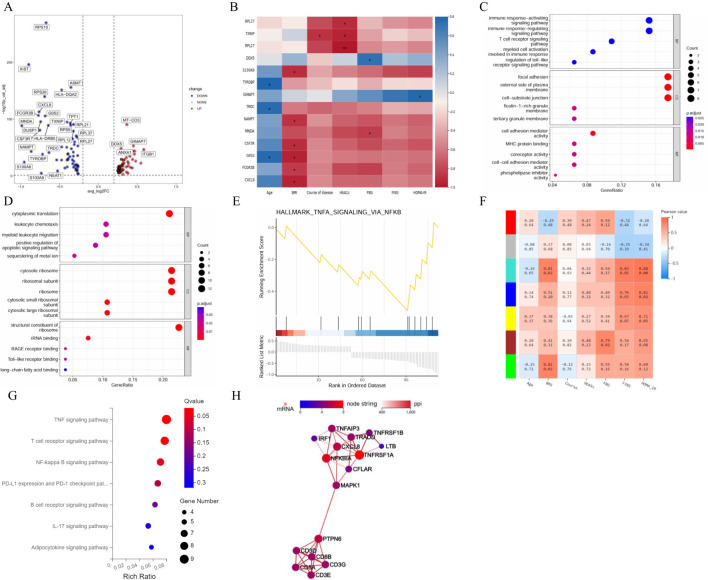
Integrated analysis of T cells. **(A)** Volcano plot showing the DEGs expressed in T cells. **(B)** Correlation analysis between DEGs and clinical characteristics. **(C)** GO enrichment analysis of upregulated marker genes. **(D)** GO enrichment analysis of downregulated marker genes. **(E)** GSEA enrichment analysis of DEGs. **(F)** Correlation analysis between clinical characteristics and each module. **(G)** KEGG pathways in the brown and turquoise modules. **(H)** Core genes by network interaction in the brown module. DEGs, differentially expressed genes; GSEA, gene set enrichment analysis; GO, Gene Ontology; KEGG, Kyoto Encyclopedia of Genes and Genomes; PPI, protein–protein interaction; BP, biological process; CC, cellular component; MF, molecular function. n=3 in each group.

The differences in the expression levels of genes between the T2DM group and the healthy group were compared to construct a volcano plot (**Figure 6A**). Among these genes, 58 were upregulated, and 61 were downregulated in T cells in the T2DM group. We then conducted a correlation analysis between the DEGs and clinical characteristics. The expression levels of RPL27, TXN1P and RPL37 were negatively correlated with HbA1c. The MNDA of genes was negatively correlated with FBG levels, and the expression levels of DDX5 were positively correlated with FBG levels. The expression levels of GIMAP7 were positively correlated with HOMA–IR levels (**Figure 6B**). GO analysis revealed that the biological process (BP) terms enriched among the DEGs were related mainly to leukocyte chemotaxis, cytoplasmic translation, positive regulation of the apoptotic signalling pathway, myeloid cell activation involved in the immune response, the T-cell receptor signalling pathway, and the immune response-regulating signalling pathway. The enriched cellular component (CC) terms were associated mainly with the cytosolic large ribosomal subunit, ribosome, cytosolic ribosome, tertiary granule membrane, and cell–substrate junction. The molecular function (MF) terms were specifically related to structural constituents of ribosome, Toll−like receptor binding, RAGE receptor binding, phospholipase inhibitor activity, and cell–cell adhesion mediator activity (**Figures 6C, D**). The first gene set, HALLMARK_TNFA_SIGNALING_VIA_NFKB, was used to generate a GSEA graph via HALLMARK pathway enrichment analysis (**Figure 6E**). The WGCNA results revealed that the genes were divided into 7 modules. According to the correlation analysis between clinical characteristics and the 7 modules, FBG levels were significantly positively correlated with the brown module. FINS and HOMA–IR levels were significantly and positively correlated with the turquoise and blue modules. With respect to the base genes with differences and the brown module, we found that HLA-DRB5, AHNAK, TYROBP and AIF1 were shared genes (**Figure 6F**). The KEGG results revealed that genes in the brown and turquoise modules were involved in the TNF signalling pathway, T-cell receptor signalling pathway and NF-κB signalling pathway (**Figure 6G**). Moreover, TNFRSF1A was the core gene in terms of network interactions in the brown module (**Figure 6H**).

**Figure 7:**
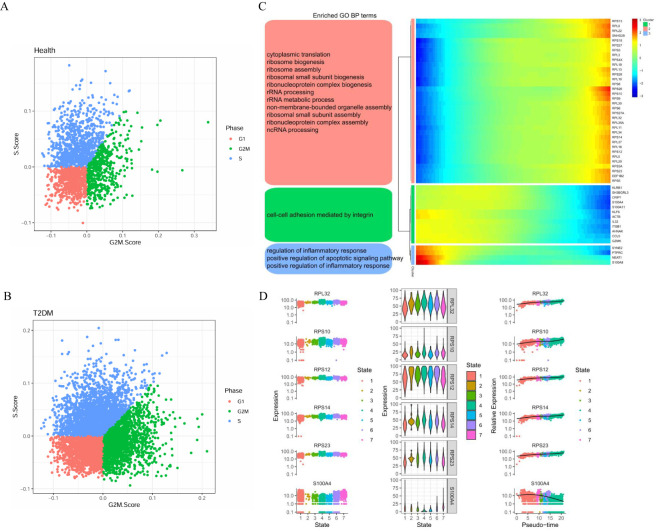
Cell cycle stages and gene expression with developmental time in T cells. **(A)** Cell cycle distribution of T cells in health group. **(B)** Cell cycle distribution of T cells in T2DM group. **(C)** Heatmap showing the dynamic gene expression of T cells and the GO analysis results. **(D)** Dynamic expression of the top genes in CD4+ T cells. T2DM, Diabetes mellitus; BP, biological process; GO, Gene Ontology. n=3 in each group.

We further analysed the cell cycle stages of T cells from healthy participants and T2DM patients. Compared with those in healthy participants, the T cells in T2DM patients were more likely to be in the G1, S and G2M states, indicating active proliferation (**Figures 7A, B**). The expression of the 50 top genes varied with developmental time. These genes are associated with cytoplasmic translation, cell–cell adhesion mediated by integrins, and regulation of the inflammatory response (**Figure 7C**). The results revealed the dynamic expression of 6 marker genes in CD4+ T cells, and different expression levels were observed at the seven stages of disease progression. The expression of the RPL32, RPS10, RPS12, RPS14 and RPS23 genes tended to increase, whereas S100A4 expression tended to decrease (**Figure 7D**).

### 3.4 Clustering and subtype analysis of monocytes

**Figure 8:**
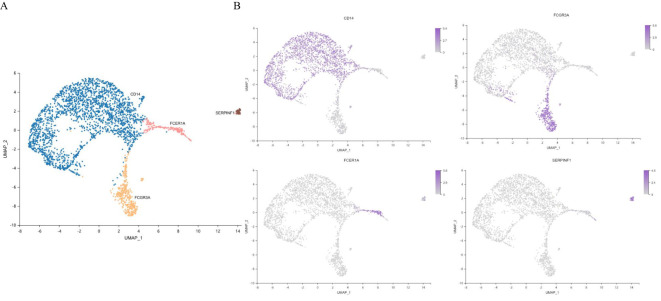
Characterization of monocytes. **(A)** UMAP plot of monocytes. **(B)** Feature map showing the marker genes for each cell type. UMAP, uniform manifold approximation and projection. n=3 in each group.

Monocytes were the most abundant nonspecific immune cells in our T2DM patient cohort. Unsupervised clustering revealed two dendritic cell clusters (including 233 cells), CD14+ monocyte clusters and FCGR3A+ monocyte clusters (including 2568 cells) (**Figure 8A**). Monocytes were annotated separately by canonical genes, and the canonical expression of genes in these cell types was consistent with the annotation (**Figure 8B**). There were 666 marker genes in monocytes and 776 in dendritic cells.

**Figure 9:**
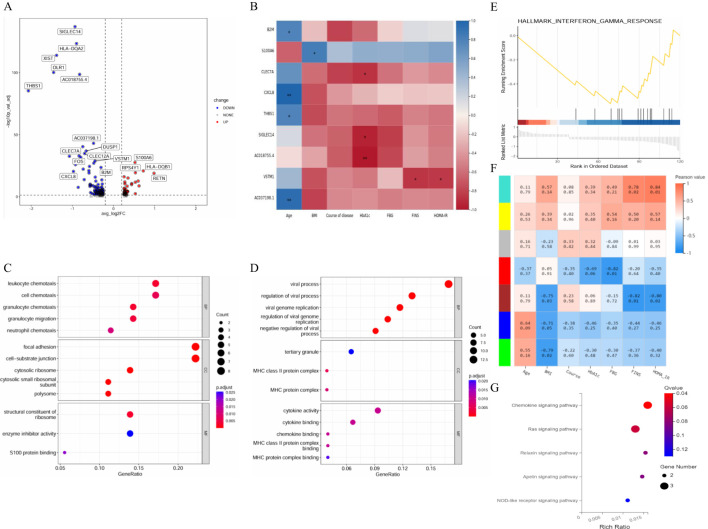
Integrated analysis of monocytes. **(A)** Volcano plot showing the DEGs in monocytes. **(B)** Correlation analysis between DEGs and clinical characteristics. **(C)** GO enrichment analysis of upregulated marker genes. **(D)** GO enrichment analysis of downregulated marker genes. **(E)** GSEA enrichment analysis of DEGs. **(F)** Correlation analysis between clinical characteristics and each module. **(G)** KEGG pathways in the red–turquoise module. DEGs, differentially expressed genes; GSEA, gene set enrichment analysis; GO, dene ontology; KEGG, Kyoto Encyclopedia of Genes and Genomes; BP, biological process; CC, cellular component; MF, molecular function. n=3 in each group.

The differences in monocyte gene expression between the T2DM group and the healthy group were compared to construct a volcano plot (**Figure 9A**). Among these genes, 51 presented upregulated expression, and 124 presented downregulated expression. The expression levels of the CLEC7A, SIGLEC14 and AC018755.4 genes were negatively correlated with HbA1c levels. The expression level of VSTM1 was negatively correlated with FINS and HOMA–IR levels (**Figure 9B**). The GO results revealed that the enriched CC terms included mainly cytosolic ribosome, cell−substrate junctions, focal adhesion and tertiary granules. The enriched MF terms were specifically related to the structural constituents of ribosome, enzyme inhibitor activity, S100 protein binding, cytokine activity and cytokine binding (**Figures 9C, D**). The first gene set, HALLMARK_INTERFERON_GAMMA_RESPONSE, was utilized to generate a GSEA graph via HALLMARK pathway enrichment analysis (**Figure 9E**). The WGCNA results revealed that the genes were divided into 7 modules. FBG levels were significantly and negatively correlated with the red module. FINS and HOMA–IR levels were significantly negatively correlated with the brown module and the turquoise module. With respect to the base DEGs and the brown module, CLEC2B, B2M and MALAT1 were identified as shared genes (**Figure 9F**). According to the KEGG results, the genes in the red module were involved in the chemokine signalling pathway (**Figure 9G**).

**Figure 10:**
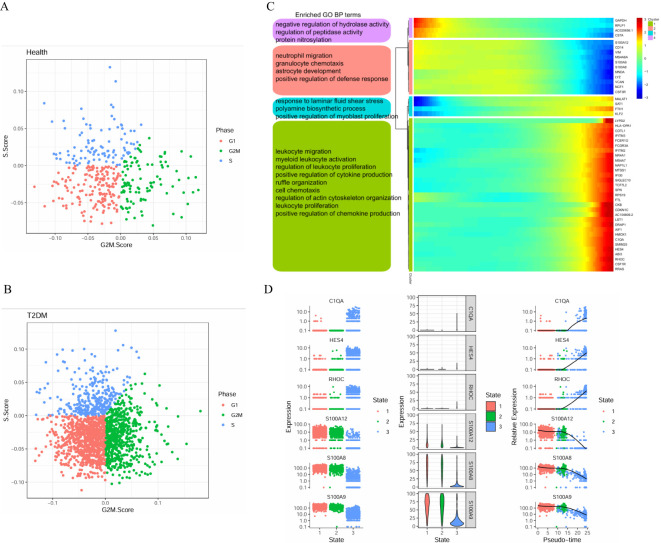
Cell cycle stages and gene expression in monocytes with developmental time. **(A)** Cell cycle distribution of monocytes in health group. **(B)** Cell cycle distribution of monocytes in T2DM group. **(C)** Heatmap showing the expression of genes related to monocytes dynamics and the GO analysis results. **(D)** Dynamic expression of the top genes in CD14+ monocytes. T2DM, Diabetes mellitus; BP, biological process; GO, Gene Ontology. n=3 in each group.

We further analysed the cell cycle stages of monocytes from healthy participants and T2DM patients. In healthy participants, monocytes were common in the G1, S and G2M states, indicating more active proliferation in T2DM patients than in healthy participants (**Figure 10A, B**). The top-50 genes whose expression varied with developmental time were associated with the negative regulation of hydrolase activity, neutrophil migration, and polyamine biosynthetic processes and the positive regulation of cytokine production (**Figure 10C**). The results revealed the dynamic expression of 6 marker genes in CD14+ monocytes, and different expression levels were observed at the three stages of disease progression. The expression levels of the C1QA, HES4 and RHOC genes tended to increase, whereas the S100A12, S100A8, and S100A9 expression levels tended to decrease (**Figure 10D**).

## 4 Discussion

Compared with T1DM status, T2DM status is associated with a greater incidence, longer duration, and greater severity of complications. scRNA-seq is widely used to characterize the basic properties of cells, and the regulation of islet cells by a subpopulation of surrounding cells has been reported in patients with DM (23, 24). Since islet cells are difficult to obtain from the human body, systematically elucidating the regulation of PBMCs in patients with T2DM is important. Previous studies have suggested that the pathogenesis of T2DM involves the immune system (8, 25).

Single-cell clustering analysis revealed that the cell clusters were annotated to 4 different cell types. On the basis of the expression levels of genes associated with endocrine and metabolic diseases, KEGG enrichment analysis revealed that these genes are involved in oxidative phosphorylation, pyrimidine metabolism, the tricarboxylic acid cycle, etc. These pathways are directly or indirectly related to the development of T2DM. Both the innate immune response and adaptive immunity are involved in inflammation. Innate immunity may cause inflammation via endogenous danger signals. Adaptive immunity also provokes inflammation via cytotoxicity, cytokines and other mediators (26). There is growing evidence supporting the idea that T2DM is a chronic inflammatory disease that results in IR and hyperglycaemia (27). In this study, the stages of T-cell activation included naïve, memory, and effector T cells. CD4+ effector T cells are the main cells that exert direct immune effects. Once activated, CD4+ effector T cells and Th1 cells exhibit many significant signs and responses to immune inflammation (28). Compared with non-T2DM patients, T2DM patients had elevated percentages of CD4+ effector T cells (29). When stimulated by antigens, memory CD4+ T cells in the peripheral blood produce effector cytokines for immune protection. A high number of memory CD4+ T cells is associated with a decreased risk of developing DM (30). Regulatory T cells play a protective role against IR in the pathogenesis of T2DM (31). The accumulation of cytotoxic CD8+ effector T cells induces inflammation and IR (32). Active circulating monocytes are inflammatory effectors that might be involved in T2DM (33). In an inflammatory state, monocytes are recruited to the affected tissue. Therefore, circulating blood monocytes levels can be used as indicators of the activation of tissue immunity (34). In addition, we also need to recognize that there are still areas that need deeper investigation. For example, which immune cells in the peripheral blood are truly involved in the destruction of pancreatic islet beta cells, which T cells or monocytes can be transported to the pancreas and affect the function of beta cells, and how pancreatic beta cells and infiltrating lymphocytes interact remain to be further studied in pancreatic samples (10).

We screened several changed candidate genes in T2DM, thus providing a reference for the study of T2DM pathogenesis. Insulin can bind to its receptor, InsR, on the cell surface and undergo a series of signalling cascades to lower blood glucose levels. For example, insulin inhibits the FoxO signalling pathway and reduces gluconeogenesis activity (35). IRS2 is an insulin substrate that regulates blood glucose levels. IRS2 knockout mice exhibit IR (36, 37). HIF-1 regulates target genes involved in inflammation, and notably, increased HIF-1 signalling induces changes in monocytes that promote the development of metabolic diseases, especially glycolysis, in the livers of T2DM patients (38–40). RPL27 expression changes in capillaries (41). Moreover, it participates in glucose and lipid metabolism (42). TXN1P is differentially expressed in patients with metabolic syndrome, which includes T2DM (43). RPL37, which encodes a ribosomal protein, is the main hub gene in DM encephalopathy and has a well-documented vasoreparative capacity (44, 45). Microvascular damage caused by sustained hyperglycaemia is correlated with MNDA (46). DDX5 is differentially expressed in obese T2DM chronic wound tissue (47). CLEC7A expression may be abnormal in DM-associated inflammation (48). SIGLEC14 enhances TNF-alpha secretion, and IL-1β release may play a role in inflammation. This effect is related to lipopolysaccharides and the NLRP3 inflammasome (49, 50). There are very few reports about AC018755.4.

Jacobi reported that low expression of HLA-DRB5 was associated with an increased risk of developing T2DM (51). AHNAK influences glucose homeostasis by regulating adipose tissue insulin sensitivity and energy expenditure (52). TYROBP is a hub gene in T2DM, especially in individuals with obesity-induced DM (53). A higher CLEC-2 concentration is a risk factor for thrombotic disease in T2DM patients (54). B2M was associated with the progression of T2DM (55). MALAT1 is a potential diagnostic biomarker for T2DM (56). According to the WGCNA network interaction results, TNFRSF1A was the core gene. Among the effector genes predicted by the Type 2 Diabetes Knowledge Portal, TNFRSF1A expression levels are positively correlated with HbA1c levels (57, 58). The mechanism by which TNFRSF1A (TNFR-1 receptor) increases T2DM susceptibility is poorly understood (59). Canagliflozin modestly decreased TNFR-1 in patients with T2DM (60). T2DM is a chronic inflammatory disease, and hyperglycaemia status and NFKB1A expression levels are closely connected (61). Among the detected genes, the expression levels of GIMAP7, HLA-DQB1 and RPL37 were related to triglyceride levels in individuals without T2DM. A previous study revealed that the causal association of triglyceride levels with DM is more obvious in young, middle-aged and nonobese people with T2DM (62). Another study investigated the relationship between HLA-DQB1 expression levels and T1DM risk and reported that HLA-DQB1 expression levels were associated with susceptibility and protective effects in T2DM patients (63). The genetic characteristics of individuals with T1DM and T2DM might include common HLA targets. HLA-DRB5 expression levels are related to T2DM status, HbA1c levels and diabetic retinopathy status, and the downregulation of HLA-DRB5 expression is associated with an increased risk of developing T2DM (51). RPL12 expression levels are related to FBG levels and BMI (64). RPS10 expression levels are related to HbA1c levels and diabetic retinopathy status (65). XIST, HLA-DQA2 and CXCL8 are common DEGs between monocytes and T cells. HLA-DQA2 expression levels are related to insulin-like growth factor (IGF) levels and neuropathy status in T2DM patients (66). HALLMARK_INTERFERON_GAMMA_RESPONSE and HALLMARK_TNFA_SIGNALING_VIA_NFKB are closely associated with the oxidative stress response (67, 68). The expression levels of genes in these pathways are significantly influenced by the occurrence and development of T2DM. Inflammatory cytokines involved in the TNFA signalling pathway regulate the insulin signalling pathway through serine phosphorylation to reduce T2DM severity (69). In addition, the T-cell receptor signalling pathway may be a pathological mechanism for GDM (70), and the T2DM phenotype of GK rats may be closely related to the T-cell receptor signalling pathway (71). Studies have revealed that the NF-κB signalling pathway is involved in the pathobiology of T2DM (72). Metformin and liraglutide effectively (beneficially) modulate immune-related NF-κB and TNFA signalling (73). The chemokine signalling pathway is involved in islet β-cell damage (74) and influences the onset and progression of T2DM (75).

The 50 genes whose expression varied with developmental time were divided into 4 clusters associated with lymphocyte-mediated immunity, protein folding, immunoglobulins and cytoplasmic translation. In T2DM patients, vascular calcification has been associated with increased S100A9 expression, which promotes the release of extracellular vesicles with a high propensity for calcification from monocytes (76). Under hyperglycaemic conditions, islets trigger an inflammatory response associated with increased expression of S100A8 (77). Research has shown that plasma S100A12 levels are higher in patients with T2DM than in patients without DM. Stepwise multiple regression analyses revealed that S100A12 may be involved in chronic inflammation in T2DM patients (78). SH3BGRL3 expression levels are closely related to IGF-1 levels. IGF-1 effectively stimulates glucose uptake into muscle tissue and increases glucose metabolism throughout the body; thus, IGF-1 can lower blood glucose levels by reducing IR (79). Increased RPS10 expression, which is driven by the maternal allele, has been shown to be a risk factor for paediatric-onset T2DM (65).

## 5 Conclusions and clinical implications

We performed scRNA-seq analysis to generate a transcriptional map of immune cells from PBMCs, thus providing a framework for understanding the immune status of T2DM patients. In addition, we explored the immune state of T cells and monocytes from many perspectives. Analysis of the target genes revealed that they were differentially expressed in each of the two groups, revealing potential key genes such as TNFRSF1A. These factors may be important in the pathogenesis and development of T2DM immunity in PBMCs. These findings may provide new insights into the treatment of T2DM. Our study also has limitations that should be noted. The sample size of this study is relatively small, and there may be some bias in the results due to factors such as the severity of the patient’s condition, large age differences, and being conducted at a single research centre. In the future, we will further validate these results through multicentre clinical trials with larger sample sizes that can also include correlation analysis with pancreatic samples from patients with T2DM. Research on the interactions of different types of immune cells may be valuable for the dissection of clinical mechanisms and treatments. Molecular biology experiments should be performed to validate the mechanisms of the genes related to immunity in T2DM. Moreover, we will identify drugs that may affect these genes and observe their clinical effects through intervention.

## Data Availability

The sequence data were obtained from the National Center for Biotechnology Information (NCBI) under the GEO accession number GSE255566. The other data are not publicly available because they contain information that could compromise the privacy of T2DM patients. Deidentified participant data supporting the published results, the study protocol, and the statistical analysis plan are available from the corresponding author reasonable request.

## Author’s note

This manuscript was previously published as a preprint at https://biorxiv.org/cgi/content/short/ 2024.01.04.574155v1. The doi is https://doi.org/10.1101/2024.01.04.574155.
